# Harmonization of Biosafety and Biosecurity Standards for High-Containment Facilities in Low- and Middle-Income Countries: An Approach From the Perspective of Occupational Safety and Health

**DOI:** 10.3389/fpubh.2019.00249

**Published:** 2019-09-12

**Authors:** Yuki Maehira, Robert C. Spencer

**Affiliations:** ^1^Department of Emerging Infectious Diseases, Nagasaki University Institute of Tropical Medicine, Nagasaki, Japan; ^2^Index Microbiology Ltd., School of Veterinary Science, University of Bristol, Bristol, United Kingdom

**Keywords:** multiple safety standards, dilemmas, low- and middle-income countries, international research and development collaboration, biosafety and biosecurity, occupational safety and health

## Abstract

Following the global-level Ebola virus disease (EVD) outbreak during 2014–2016, international collaboration with multiorganizational participation has rapidly increased. Given the greater priorities for research and development (R&D) outcomes despite the quantitative and qualitative lack of high-containment laboratory facilities in low- and middle-income countries (LMICs), where biological targets for investigation are located near their natural habitats, occupational readiness for health workers' safety has not been well-addressed, where limited global expert human resources are being deployed to high-containment laboratories including biosafety level 4 (BSL-4) facilities for case management and medical investigations. Pursuing scientific and managerial success to make laboratories efficient and productive, most laboratory safety policies have focused on the functionality of technical skills or performance, procedural methodologies, and supervision over the employees to collaborate in LMICs. The experts dispatched from advanced countries bring a long list of scientific tasks with high-tech devices, supplies, and training programs to introduce their collaboration with local partners in LMICs. However, the dispatched experts would subsequently realize their list becomes endless to establish their basic functions required in high-containment laboratories to ensure qualified scientific outcomes in LMICs. Under such circumstances where dual or multiple policies and standards accommodated pose dilemmas for operational procedures to ensure biosafety and biosecurity, all the frontline experts from both LMICs and advanced countries may be exposed to significant risks of life-threating infection of highly pathogenic agents like EVD, without any pragmatic measures or road maps to establish valued international collaboration, pursuing its sustainability. Given the fact mentioned above, we conducted a quick review of the key biosafety and biosecurity management documents, relevant policy analyses, and research to understand the current status and, if any, measures to dissolve critical dilemmas mentioned above. As a result, we found that occupational safety and health (OSH) aspects had not been sufficiently addressed, particularly in the context of international BSL-4 collaboration in LMICs. Moreover, consideration of OSH can be one of the key drivers to make such collaborative interventions more pragmatic, safer to reorient, harness disease-based vertical approaches, and harmonize policies of biosafety and biosecurity, particularly for collaborations organized in resource-limited settings.

## Introduction

Various international standards and guidelines are available for licensing or accreditation of institutional technological skills and performance quality management for laboratory research and development (R&D) of infectious pathogens, including the World Health Organization (WHO) items (1, 2), with a general consensus to enhance laboratory medicine and biological and medical research, globally. These regularly refer to Good Laboratory Practice (GLP), International Organization for Standardization (ISO) credentials, Clinical and Laboratory Standards Institute (CLSI) requirements, or the Organization for Economic Cooperation and Development (OECD) GLP principles. In particular for biosafety level 4 (BSL-4) investigation, guidelines for biosafety and biosecurity control have been developed on similar concepts to advance technological capacity by installing devices and enhancing technical performance and skills building for local experts (3–6), as targeted in the guidelines for laboratory quality management (1), in line with the threat level of pathogens categorized on each virulence and epidemiological characteristics of microorganism (3).

For this paper, in line with the definitions offered by the Laboratory Biorisk Management guidelines agreed in the EU in 2011(CWA 15793) (7), “biosafety” is defined as the containment principles, technologies, and practices that are implemented to prevent unintentional exposure to biological agents and toxins or their accidental release, while “biosecurity” is defined in CWA 15793 as the protection, control of, and accountability for biological agents and toxins within laboratories, in order to prevent their loss, theft, misuse, diversion, unauthorized access, or intentional unauthorized release.

Occupational safety and health (OSH) are the disciplines dealing with the prevention of work-related ill health as well as the promotion of health and welfare for workers (8) as an integral component to support the performance quality standards of laboratory investigations. Since 2018, those have been evaluated in reference to ISO 45001 (9), integrating the key predecessor Occupational Health and Safety Assessment Series (OHSAS) 18001 developed in 2007 (10).

From a bioethical standpoint, the principles of the Helsinki Declaration (11) or the Belmont Report (12) have been developed with an exclusive focus on human rights protection for the patients/target sample populations from planning to implementation, addressing confidentiality to use personal information in the study process and results. Although the Helsinki Declaration requires the qualified profession by extension of the articles that protect research subjects from unethical conduct of medical R&D projects including those of international collaboration led by the diverse experts, there is no clarity regarding the inclusion of the OSH rights of R&D implementers (researchers or health experts) for their protection from infection risks, particularly during the outbreaks of deadly diseases.

Without proper facilities and adherence to safety protocols, emerging issues related to laboratory medicine and investigational R&D may violate the ethical sphere of OSH (13). Recently, the guidelines contextualizing OSH have been evolving in the domain of highly pathogenic emerging infectious diseases control (3, 14, 15). However, those tools reiterate conventional norms of engineering control for physical isolation of specific hazards and biotechnological performance, requiring further adherence to sanitary precautions and hygiene procedures for infection prevention and control (IPC). However, OSH issues for BSL-4 workforces cannot be discussed only on the level of isolated technical solutions (16), relying heavily on the appropriateness of the donning and doffing of personal protection equipment (PPE) for universal adaptation (17).

To date, the OSH norms or regulations applied to international BSL-4 R&D coordination have not been actively discussed to provide practical solutions to guide and protect both local and foreign healthcare workers or researchers equitably. To ensure performance quality for the laboratories and medical facilities in low- and middle-income countries (LMICs), particularly in the context of international BSL-4 R&D collaboration, we addressed the lack of OSH approaches to raise the question as to whether appropriate OSH capacity prequalification can be the key driver to enable further ethical R&D coordination for biosafety and biosecurity preparedness.

## Methods of Analytical Review

The main aim of this review is to identify the potential barriers to integrating OSH concepts for health experts and researchers in the biosafety and biosecurity management in the practice of international BSL-4 R&D collaboration in LMICs and thereby determine what are missing to resolve the dilemmas we have encountered in attempting to ensure prequalified basic infection risk control skills and technical performance of work with our diverse local partners for implementation of international R&D collaboration in LMICs.

**Figure 1:**
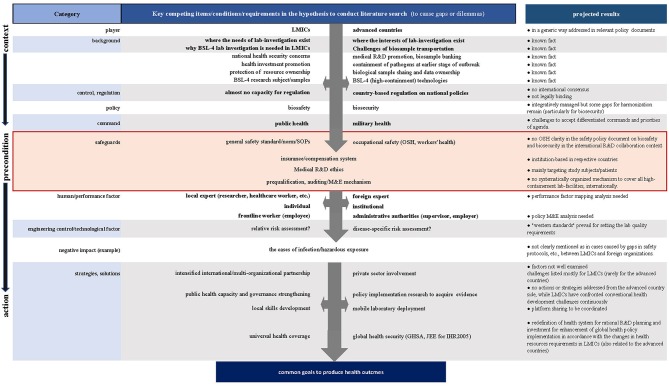
Flow diagram of a hypothesis-based stepwise literature search.

To achieve this aim, we adopted a stepwise approach on preset hypotheses based on our projected literature search results (Figure 1), for which our experimental analysis of various international BSL-4 R&D settings in LMICs was taken into consideration.

First, we reexamined the backgrounds and preconditions where we presumed the gaps or dilemmas to manage field R&D operations. Second, we contrasted potential risk scenarios to compare the competing interests, procedures, or modalities underlying international R&D collaboration to identify drivers or barriers. We also looked at the negative impacts evidenced in the case reports to assess probable causes or background to increase healthcare workers' infection and security breaches or mishaps, if any, in the research experts.

We searched for and reviewed potentially eligible information sources, including international and regional guidelines, tools for capacity assessment, and documents for standardization or harmonization, particularly for BSL-4 laboratory management, prioritizing those applied to the collaboration in LMICs. We also assessed relevant OSH regulatory items, including peer-reviewed academic literature databases, organizational web portals, and relevant policy research. All those processed as mentioned above enabled us to retrieve the latest information, particularly after the 2014–2016 Ebola virus disease (EVD) outbreak in West Africa, as well as the information that might have an impact on key safety policy development. Simultaneously, we attempted to compare relevant information resources endorsed before and after the 2014–2016 EVD outbreak in West Africa. We therefore predominantly collected information sources from the period 2000–2018, assuming probable changes in policies, guidelines, or regulatory trends from different viewpoints to evaluate medical R&D practices for biosafety and biosecurity, especially for BSL-4 investigational operations in LMICs.

The key WHO documents related to biosafety and biosecurity—including those for WHO-International Health Regulations (IHR) (2005) and the Joint External Evaluation (JEE) tool, as well as the Global Health Security Agenda (GHSA) web portal—were reviewed to see whether international R&D contexts had been addressed or reflected to examine the changes in global health security trends and relevant policies.

Supporting literatures were searched to collect the following: case reports or systematic reviews of infection risk exposure, and case reports and analysis in the contexts of both advanced and developing countries, including the guiding documents for safety precautions for the major international medical non-governmental organizations (NGOs).

The search strategy to combine keywords and MeSH (Medical Subject Headings) was conducted using MEDLINE, the Cochrane database of systematic review, built into PubMed syntax. EMBASE, ProQuest, and the Google Search Engine, books, conference proceedings, and key declarations, and global health initiatives were also searched, with the view to identify the main areas of debate as well as to provide supportive background information.

## Search Results

From the literature search, no systematic reviews, evidence-based policy implementation analyses, or comparative policy studies on high-containment laboratory management in LMICs and advanced countries were identified. Subsequently, 144 non-comparative articles were retrieved: 33 items focusing advanced country's settings, 22 items in the developing country's context, 25 items with global-level viewpoints, and 35 WHO items to provide background and reference information for our question and points for argument. We reviewed relevant guidance documents including the codes of conduct, standard operating procedures (SOPs), standardization tools, accreditation or certification mechanisms, and OSH norms and bioethics to protect human rights and security.

Selected key global policy and guideline documents were assessed chronologically to overview and compare the descriptions that address or imply international R&D contexts as well as the OSH aspects, attempting to provide evidences of some changes made after the EVD outbreak during 2014–2016 (Supplemental Table 1).

Since the experts' concerns emerged to clarify challenges with regard to the underlying global expansion of high-containment biological laboratories (18), with reference to the 2011 WHO Guidance Document for Responsible Life Science Research for Global Health Security (19), it is notable that the WHO introduced the Guidance for Managing Ethical Issues in Infectious Diseases Outbreaks in 2016 (20) as part of their response to the EVD outbreak in West Africa, which addressed the OSH pre- and postdeployment conditions for frontline workers including researchers in the context of public health ethics. It laid down the principles of equity and transparency among responsible entities for assignment of their workers, addressing that “risks are distributed among individuals and occupational categories in an equitable manner.” Those items imply the settings for international collaboration, although it does not provide specific guidance for BSL-4 investigations while requiring transparency and accountability for trusteeship among the concerned entities.

Despite the efforts made to establish globally applicable technical standards and guidelines, most of them do not clearly address or consider the points relating to international BSL-4 collaboration in developing their underlying principles for high-containment laboratory quality management. Relevant descriptions in the documents assessed are in generalized ways to encourage better relationship for trust and successful coordination of technical outcomes, paying attention to each worker's obligation and rights to produce his or her scientific results, considering how to demonstrate and organize local ownership to manage biological samples and data. The only exception is in the descriptions on the “Respond” actions for the indicators of “Medical countermeasures and personnel deployment” in the WHO JEE tool (2nd Edition, 2018), where we see relatively clear outlines related to OSH instructions for the establishment of qualified international collaboration and coordination of personnel, although participation in this JEE framework is on a voluntary basis (21).

As shown in Figure 1, OSH can be recognized as the key precondition to progress biosafety and biosecurity management toward the expected scientific and technical performance of works in laboratories regardless of their locations for works. However, any guidance cannot be concretely identified in terms of OSH aspects; moreover, there is as yet no global consensus regarding systematically coordinated standards or mechanisms to provide pragmatic clarity about high-containment laboratory management applicable to the growing numbers of international research and R&D collaboration, such as in projects under the WHO Blueprint (22) or Monitored Emergency Use of Unregistered and Investigational Interventions (MEURI) (23) initiatives.

## Descriptive Analysis for Discussions

### Illustration of the Potential Risks at High-Containment Laboratory Workplaces Under Safety Controls With Dual or Multiple Standards

The key health technology resources are distant from advanced countries, while clinical sample availability of the diseases we need to control is localized because of disease endemicity in LMICs (24).

The International Air Transportation Association (IATA) Dangerous Good Regulations Shipping is in place to exchange or share infectious agents. Complications associated with such transportation of infectious agents have seen the debate move in the direction of favoring the development of high-containment laboratories closer to the epicenters. However, this requires financial and infrastructure support not just in facility maintenance but also in personnel training to ensure safe and secured scientific outcomes.

In such processes of investigational health resource deployment, foreign healthcare workers often play a key role in leading and coordinating medical case management and supporting local health authorities by being integrated into the local teams. In any designated health facility for collaboration, a variety of frontline experts must work together, observing their own laboratory safety policies. Such circumstance may allow two or more different safety management policies to coexist to regulate tasks and workers at the same time and in the same place, in order thereby to increase the level of complexity (25). Likewise, we often confront technological and procedural conflicts in the application of both local and foreign safety protocols. Such procedural conflicts may largely affect safety performance of work at BSL-4.

Laboratory safety management can be implemented by the establishment of a safety policy, where employers and employees share and recognize the roles and responsibilities of all experts who collaborate in a laboratory, to follow certain work procedures and methods based on a mutual consensus for health protection, primarily on an institutional basis. Partner organizations may recognize their own OSH protocol, if available, and regulations that have been set up differently. In other words, foreign workforces need to ensure their safety, based on their home organization's policy, while the local workforces manage their practices in line with their conventional rules observed at their own institution for safety, if available. As such, the frequent discrepancy in safety administration systems between local and foreign policies to control biosafety and biosecurity may influence to increase infection risks for the experts due to the gaps encountered in each work and safety procedure, despite their observance of each general precaution or guideline.

Modern global practices recommend respecting local standards or norms to demonstrate sensibility and courtesy to the local medical administration in health development contexts for assistance provided for LMICs. The global health security authorities have encouraged development of investigational capacity for clinical practices and medical R&D in the laboratories to address the unmet health needs of LMICs. As a result, the world has become better prepared in dealing with and managing outbreaks and epidemics of emerging life-threatening infectious diseases. On the donor or investor side in the R&D partnership, mainly led by the advanced countries, valuable efforts have been made to build many international biological threat reduction programs such as GHSA, IHR, One Health, and the recommendations to review the WHO Laboratory Biosafety Manual [3]. The International Federation of Biosafety Association (IFBA) works through establishment of its bioengineering working group, which explores practical and sustainable solutions for high-containment laboratories to manage dangerous pathogens in LMICs (26, 27). For African countries, Strengthening Laboratory Management Toward Accreditation (SLMTA) (28, 29) and the WHO Guide for the Stepwise Laboratory Improvement Process Toward Accreditation in the African Region (SLIPTA) (30) are available to demonstrate its pragmatic approach, which is measurable to assess LMICs' preparedness and response functionalities to enhance positive, self-directed laboratory quality management for local practices. In the SLMTA framework, a mentoring partnership mechanism introduced for some African countries with the globally accredited research institutes (29) could provide a good model for the furtherance of innovative international biosafety and biosecurity harmonization.

However, those capacity development platforms do not cover OSH sufficiently in an international R&D collaboration context.

### The Dilemmas Encountered in Practicing BSL-4 International R&D Collaboration in LMICs

Following laboratory infection incidents (31) resulting in a call for a biological safety regulatory framework, BSL-4 facilities emerged in the United States (32) and Europe (33). Laboratory safety management and OSH coordination for international collaboration are mostly intangible (34). However, the debates on the need for regulation or harmonization in research and clinical studies, particularly on highly pathogenic infectious diseases, are quite limited among experts at the global level (18, 35).

In the reality of working with local partners in LMICs, diverse ethical dilemmas will inevitably be encountered, as the examples described below, when the concerned experts would need to accommodate multiple standards for technological capacity transition in LMICs in pursing common goals for the containment of biological threats as a shared global health security concern.

The first dilemma concerns whether there is a regulatory conflict in accepting laboratory buildings and facility infrastructure are utilized in LMICs by personnel from those advanced countries in which such buildings or facilities would not be accepted.

As an integral component of local public health response mechanism, mobile laboratories can be deployed in LMICs (36). It is a unique modality whereby advanced countries can ensure a safer and secure work environment on their own standards, observing their own national safety policy as much as possible. Pursuing point-of-care diagnosis mechanisms and with access to the biological agents for investigational research and validation of new diagnostic assays, a mobile laboratory can provide invaluable opportunities with the minimum equipment essential to the laboratories in resource-limited setting of LMICs, which can be broken down into modules for ease of transport, although this option lacks permanency in local health systems. Not having to rely on local facilities where infection risk factors may be uncertain, the foreign experts can work exclusively on their own operational procedures, separately from the local collaborators. However, it cannot rule out ethical concerns for R&D collaboration and OSH issues in LMICs. Deployment of mobile laboratories would require different rules and regulations to manage OSH for workers to collaborate, clarifying its uniqueness, limitations, or competencies to reorganize each role played by local and foreign experts.

In addition to the physical, infrastructure problems mentioned above, persisting major technology gaps between the facilities in advanced nations and those in LMICs constitute the second dilemma in attempting to control infection risks. Despite the enormous efforts of experts in LMICs to meet the international standards in the key guidance documents (2, 37, 38) for device installation specifications, there are critical challenges in resource-limited circumstances to explore logistics service channels for sustainability. Moreover, it is not appropriate to transport the laboratory devices that had been used in the high-containment facilities, particularly at BSL-4, to the outside for maintenance as they are regarded as contaminated.

Sustainable investment in advanced technologies is challenging to maintain its quality and function for confirmatory diagnosis of and medical research on deadly contagious pathogens, such as Marburg and Ebola viruses in LMICs. While maintaining reasonable risk–return tradeoff options for investigation of such irreplaceable biological resources for advancement of research to develop much-needed medical countermeasures (34), infection risks to access such dangerous pathogens should be properly regulated for maintenance of the facility and devices.

At the same time, the concerned experts should be more pragmatic in considering whether the current biomedical and molecular techniques for “western standard” technologies are all required in LMICs, and whether there is scientific evidence that all of these physical risk isolation facilities, devices, and management systems are all necessary to reduce work-related procedural discomfort and ensure OSH.

The third dilemma relates to technical performance—the human behavioral factors from which we need to learn and train each other for the attainment of mutually agreeable levels of work to cover the knowledge about disease transmission and viral adversaries. However, those technological and performance factors might be primarily evaluated from scientific result-based viewpoints to generate outputs through collaboration without taking into consideration the underlying regulatory ethics for OSH equity, and the intricacies of developing sound operational procedures by identifying administrative, structural weakness for laboratories in LMICs. Also, such skills and performance qualification standards may vary even among the foreign institutes, partly because of their insufficient lab investigation experiences particularly in LMICs. While capacity strengthening is required more for LMICs than for their foreign partners, outbreaks of EVD or other viral hemorrhagic fevers will almost certainly overwhelm local health systems without putting into place the robust pathology and laboratory medicine in LMICs (39, 40).

For foreign institutions, psychological factors, respectfulness of local ownership, and a mindset attuned to following local practices sometimes do not work positively in the introductory phase of risk management and IPC collaboration.

Local experts on the ground may also be confident of their own systems and sometimes blindly do the bidding only of their own authorities, which may, albeit unconsciously, place their collaborators at risk. A similar context in which infection risks are encountered arises particularly at the beginning of a collaboration arrangement: habitual practices and norms based on beliefs, under- or overestimation, or complacency (25) for both local and foreign authorities often present challenges in providing care for patients or managing infectious sample operations in line with their own institutional administration systems. Risks can be significantly increased in the transitional phase of establishing collaborative laboratory investigation mechanisms. Such phases may last longer than what they estimated being influenced by unpredictable factors involved in unstable R&D administration and regulatory policies.

As is often the case, civil–military coordination of international public health responses would make OSH environments more complex in terms of managing different safety and security SOPs among the organizations (41–43).

To deal with deadly diseases such as EVD, Marburg, or Lassa virus infection, health experts or clinical researchers may take risks for scientific benefit to access such hazardous agents. Such practices would jeopardize the laboratory or healthcare facility's OSH policies that act as a hedge against infection risks. Proponents of such risk–benefit tradeoff under multiple OSH policies may argue that acceptance of higher workplace hazards of infection is defensible because the workers are experts by nature who should have enough specialized knowledge and skills to consent to manage the risks involved in performing dangerous work for the agreed-on wage.

Interlinked with the third dilemma, the fourth dilemma concerns whether those in collaboration can ethically argue for accepting differentiated dual standards for employer safeguards or health insurance systems that have been established and applied in each country's OSH policy. It is evident that we, the experts, have all been doing this without rigorous analysis since international R&D and public health collaboration began, while global health security authorities doubt that dual standards with two or more sets of laboratory biosafety and biosecurity rules can be functional (44). It is also the issue as to whether such dual-safety insurance systems could facilitate mutual trust for performance among diverse experts in the laboratories where workers are equally exposed to infection hazards and risks in LMICs.

### Holistic Laboratory Safety Control Supported by Skilled Work Performance

The “risky bravado of the hot laboratory” has become a trend where advanced countries competitively demonstrate their “international prestige and technical prowess” in relation to medicinal and medicotechnical devices or equipment (45) to ensure safety for working with dangerous agents in healthcare facilities in LMICs. This has led to risk reduction becoming more expensive, as the overheated global competition for “hot-lab” technology development now requires a virtual zero tolerance of infectious risks in a state-of-art laboratory dealing with BSL-4 pathogens.

The safety regulations to control exposure to infectious agents are on a different footing from those for radiation control, where the dose limitation principle is applied (46). Then, can we answer the question of exactly which levels of safety or security thresholds should be technically pursued for IPC in LMICs? Is current procedure for pretreatment of blood or tissue samples sufficiently safe for further use in molecular detection strategies when live agents are in the equation? What measures can be selectively applied within our limited resources to observe such hazardous exposure limits? Although our OSH objectives are set to minimize infection risks and health hazards, we all know that we cannot achieve zero risk. We also know that engineering control and performance improvement cannot create entirely safe and secured working conditions, as the examples of the incidences reported even in advanced countries (47–49). This is partly because that the norms vary a great deal among the professions for medical services delivery based on each specialty. Likewise, there are the gaps between professional norms for public health and emergency medical responses, and those for investigational R&D. One US expert of biosafety and biosecurity pointed out that the reason for these gaps “is the lack of a safety culture” worldwide rather than “a lack of knowledge or training, or even a lack of engineering resources (50).” Those demands listed by both LMICs and the foreign partner organizations usually prioritize physical safeguards in the installation of equipment and devices, pursuing the “Western standards” led by the developed countries (51). The list of requirements for BSL-4 capacity remains endless, perpetually addressing the complex adaptive process to ensure a substantial impact in improving health systems' responsiveness.

Therefore, we must raise the question of whether all those demands have been scientifically proven as necessary and essential for high-containment facilities, particularly in LMICs. In other words, we question whether there are any scientifically evaluated reasons for requiring installation of all those in the list developed with the view of advanced countries, where different risk assessment standards and factors apply. It is true that we cannot wait until basic conditions in LMICs are ready by themselves under current circumstance where there are no regulations or oversight to determine what should be prepared otherwise than SLIPTA to audit the progress (52).

Strengthening skills as a bottom-up strategy for LMICs' lab capacity building is one of the paramount concerns among global health authorities and experts. There is no doubt that attempting to improve professional knowledge and skills is crucial as performance factors to maximize the potential of devices and human resources for safer, secured laboratory work. Moreover, the practices of laboratory workers should be reasonably monitored, evaluated, and justified to produce safety benefits to public health experts at a higher risk of infection than the general public, particularly in dealing with deadly infectious agents.

In addition to the normative needs (53) laid down for the developing partners, there are expressed needs that beneficiary countries require to build and strengthen their national health systems for enabling their strategic mobilization of resources to monitor relevant processes for control of the disease epidemics hopefully under their ownership. However, a recent report found that stakeholder involvement from LMICs is persistently underrepresented to raise their concerns for addressing specific local preconditions in own countries to the GHSA toward the normative consensus (5).

### Global Trend Toward a Horizontal, Universal Health Development Approach

“Global health security” is a loaded term, although the concept has progressively been translated from the conventional intelligence of medical sciences in a geopolitical context to more generalized solutions for population health development in a broader sense. Today, ever-changing biogeographical modalities and epidemiological dynamics of infectious diseases are interlinked with globalization factors that include rapid population growth, unplanned urbanization, migration due to various conflicts, and environmental catastrophe. The authorities concerned therefore must reshape our agenda and prioritize sustainability with strategic resource allocation for own survival. Currently, global health security and universal health coverage (UHC) are interpreted as the “two sides of one coin (54, 55).” The concept of UHC is interwoven with health-related targets in the sustainable development goal (SDG) framework (56).

Although not expressly characterized in the SDG framework, the GHSA (57) project has been actively implemented in LMICs, and other health surveillance capacity development programs such as the Regional Disease Surveillance Systems Enhancement (REDISSE) projects (58) by the World Bank group or the Connecting Organizations for Regional Diseases Surveillance (CORDS) (59) are implementing activities to make direct interventions in various partnership modalities with LMICs. This goes beyond the national, organizational borders of biosafety and biosecurity control frameworks, and high-containment laboratory capacity development is an integral part of ensuring early detection of outbreaks for the containment of epidemic-prone diseases in LMICs. However, again, central tracking authorities are currently absent from BSL-4 facilities around the world (52).

As a critical mechanism to supplement such global health security functionality, the JEE tool for IHR (2005) takes preventive approaches to promote the strengthening of the eight core capacities of health systems: national legislation, policy and financial mechanisms, coordination with national focal points, risk communications, surveillance for response, preparedness, human resource, and laboratory networking. Here, biosafety and biosecurity issues are described as prevention measures, and national laboratory systems development is required for the detection of outbreaks (60).

For the establishment of national biosafety and biosecurity systems, it is necessary for the legal embedding of laboratory capacity and relevant oversight systems at the national level in order to manage dangerous pathogens. However, the JEE mechanism can only function with voluntary participation, focusing on strengthening national level health systems for improved responsiveness to global health security issues while implying key biosafety and biosecurity issues that usually involve foreign organizations' interventions for long-term preparedness and planning.

This omission, however, may simply be because the concerned authorities have considered the OSH does not need to be articulated. They may probably consider that it should be naturally covered under the current R&D safety policy umbrella. Or, they think such collaborative assistance for capacity strengthening may be performed as a transitional or temporary component for intervention made only as a contingency setting, therefore not particularly well-aligned with approaches to strengthening a sustainable health system. Meanwhile, recent analytical reports (61, 62) addressed the fact that the JEE demonstrates critical capability weaknesses in areas vital for the operation of biosafety and biosecurity programs, highlighting shortcomings or assistance gaps in the coordination of external and domestic planning and improvement of biosecurity and biosafety capabilities.

Embedding the IHR mechanism in the conceptual framework of global health security, GHSA emphasizes health system capacity development, including field surveillance capacity for enabling earlier detection and containment of epidemic-prone indexes while minimizing potential damage in countries at various developmental statuses. As a broader concept embracing global health security, along with the IHR and GHSA, the UHC initiative has enlarged our multisectoral vision of primary health care (PHC), emphasizing universality of health equity for service delivery to individuals based on unmet community and patient-centered needs. The UHC encompasses all facets to mobilize potential solutions for disease prevention and control to cover all health development aspects identified in both advanced countries and LMICs based on the progress of the millennium development goals (MDGs) (63) toward attaining SDGs.

### The Value of Public–Private Partnership

Supported by various international health development initiatives as mentioned above, private sector engagement becomes visible and active in various global action frameworks, including GHSA (64) and UHC (65, 66), participating in the global expansion of civil society involvement encouraged since the PHC Alma-Ata declaration (67).

To support international resource matching efforts, some useful measures have been introduced for private industries or other non-state actors: the WHO Service Availability and Readiness Assessment (SARA) (68) and the Strategic Partnership Portal for IHR (2005) and Health Security (SPH) (69), and those reference sources can provide regular updates and rationales for each country's needs to demonstrate financial accountability to their investing partners. The WHO initiative for a compendium of innovative health technologies for low-resource settings (70) and the activities to develop preferred product characteristics (PPC) and target product profiles (TPPs) for new vaccines (71) or PPE products (72) are highly useful platforms for involving the private sector.

Those initiatives have created pragmatic health technology production domains that often do not follow the high-tech path, but attempt to reevaluate and apply readily available technology resources, materials, and devices in innovatively different ways as health solutions for LMICs, from different viewpoints for usability, toughness, simplicity, user-friendliness, substitutability, and cost–performance for a sustainability applicable to the working conditions in LMICs. Technological intervention collaboration is not an easy task for academic and public organizations to achieve by themselves; thus, private sector participation is the key. The private sector is one key component of the non-state actors (73) who can contribute supporting global public health preparedness and responses. Some examples of medicotechnical resources such as portable nanotechnology for DNA/RNA sequencing (74), rapid diagnostics (75), and IPC tools (76) have been developed through various partnerships including the public–private R&D collaborations and are already available for deployment to control epidemics.

However, the private sector often faces challenges in a limited and regulated market where they can contribute to global health security in reducing costs to an affordable level for LMICs and establishing a sustainable logistics infrastructure. If any development support would not be available along with options of insurance or subsidy programs by the public sector, the private sector itself cannot sufficiently manage their service delivery, particularly under their coordination with the state-level actors to collaborate for outreach marketing and reasonable supply of such public goods to LMICs.

Importantly, those innovative partnerships would encourage the private industries and technology development organizations to collaborate more in LMICs, involving local venders. This implies that additional safety considerations would therefore be required for the risks faced by these non-healthcare service providers who might be at risk of exposure to infectious agents (25, 29).

## Recommendations

In light of the dramatic changes observed in occupational environment dynamics, all authorities and experts concerned must encourage open discussions and explore constructive measures that can reorganize and rationalize the safety, security, and sustainability of field-based international R&D collaboration. Especially as urgent tasks for the BSL-4 investigations required to involve high-threat pathogens in resource-limited settings in LMICs, local and foreign health experts need to involve their own employers at the institutional level in their discussions for partnership coordination more actively than before, to aspirate useful ideas or share their concerns, and to develop commonly agreed, adaptive safety and security standards and guidelines specifically for operational settings of their international R&D collaboration.

In this paper, we raise the question as to whether OSH prequalification or regulatory capacity M&E mechanisms can be pursued simultaneously with biosafety and biosecurity preparedness to conduct BSL-4 investigations or establish BSL-4 facilities in the international frameworks for collaboration. To enlarge and deepen relevant discussions, we addressed the points below regarding question or dilemmas in this review paper:

▪ Is it ethically acceptable or appropriate to work in lab conditions in LMICs that do not meet the safety standards followed in advanced countries such as European, American, and Japanese BSL-4 facilities where foreign experts have been trained?
▪ Should all concerned experts accept or compromise the lower safety standards that can only be operated in LMICs in order to make a lab facility available to tackle common public health threats during outbreaks?
▪ If not acceptable, then how can all concerned parties identify the procedure agreeable or adaptive in collaboration to resolve the problems of dual or multiple safety standards and SOPs for collaboration in the same facility?

**Figure 2:**
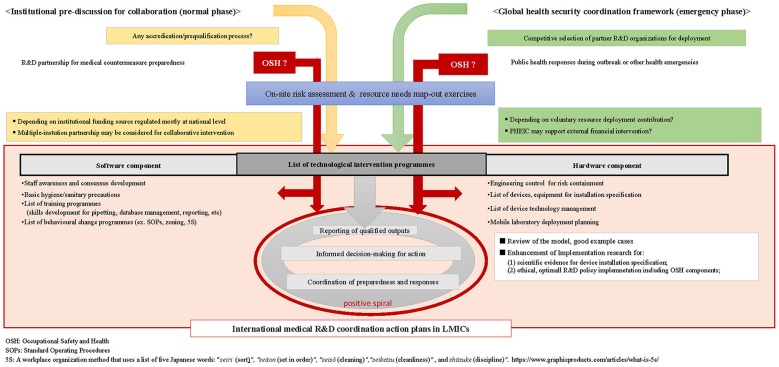
Flow diagram of generic international medical and public health R&D preparedness and response collaboration.

Strategic OSH policy integration should be one of the key preconditions for all who collaborate, regardless of the origins of individual experts or positions in the financial hierarchy. To reach the agreeable procedures among all collaborating parties, analytical integration of the OSH context should supersede the lists of technical intervention items, as suggested in Figure 2.

First, the current OSH for high-containment laboratory management should be reexamined by foreign parties in advanced countries to redetermine their preconditions for sustainable and rationalized resource deployment. By acquiring evidences to discuss with employers, OSH policy decision makers, or R&D donors, the experts must know whether such safeguards or OSH policies on international R&D collaboration are available and properly applicable to accord with the conditions for collaborating partners in LMICs. All concerned experts need to develop model cases to support prioritization and selection for device installation specification within the LMICs context with a systematic viewpoint to ensure their implementation feasibility and sustainability.

For publicly funded international R&D project management in particular, national-level discussions are needed to promote commonly adaptive regulatory, M&E mechanism that would not impose unnecessary restrictions to enhance international medical R&D collaboration without discouraging efforts of the concerned authorities for medicotechnical advancement.

Mechanisms to monitor high-containment facilities in a mentoring partnership with foreign research organizations can potentially generate positive outcomes for LMICs, as demonstrated through the systems of SLMTA or SLIPTA (28, 30). This entails that the partnering foreign research organizations would have to be prequalified for such mentoring capacity development and resource allocation strategies to enhance international collaboration.

In this regard, global health security experts and authorities concerned should suggest development of appropriate adaptive mechanism for institutional prequalification or accreditation to normative mechanisms through appropriate channels, such as those available through WHO. These efforts can be synchronized with enhancement of further in-depth policy analysis through enhancement of implementation research (77) in line with global efforts for updating the WHO Biosafety Manual, which has been suggested among the experts (3).

By enhancing policy implementation research, conditional factors and norms can be mapped out for further contextual understanding of specific situations—differentiating the positions of researchers, laboratory technicians, healthcare providers, or other support staff—identifying where ethical conflicts of biosafety and biosecurity policy may be incurred that are interrelated with OSH policies developed on different viewpoints for risk analysis.

In other words, unless presently available resources can be clearly mapped out for the expected collaboration at specific high-containment facilities along with individual purposes and roles for the missions, lists of essential resources and the operational roadmap cannot be determined or justified to plan the activities for the needs to facilitate BSL-4 laboratory mechanisms in LMICs. In this regard, health systems development is pivotal to forge a global-level consensus for refining the definition of health systems (78) in order to facilitate the synergic advancement of global health security and UHC to harness the relevant vertical strategies in furtherance of public health objectives for control of BSL-4 diseases outbreak.

Biosafety and biosecurity principles should be more practical and more equitable in order to ensure OSH for both local and foreign experts, as basic human rights for health protection, regardless of the stage of consensus development to establish collaboration. An ethically informed, adaptive OSH policy development as well as harmonization of biosafety and biosecurity are neither easy nor simple. However, the standards for OSH management should be pursued on a single, commonly adopted policy, for which a detailed factual and normative analysis of institutional guidelines and regulations can provide pragmatic insights. Also, the discussion process itself will facilitate positive engagement of all concerned to ensure OSH for the limited number of BSL-4 resources as well as to manage surge capacity systematically for global responses to multiple outbreak incidents occurring concurrently.

Recognizing OSH prepositioning is important as a key rate-limiting factor for BSL-4 fulfillment of technological and administrative interventions, collaborative partnerships can be enhanced, ensuring a positive spiral for progress in discussion of safety assurance. Taking efforts to optimize both technical and behavioral regulatory safety requirement thresholds, operational roadmaps can be defined. Moreover, these efforts should be on a shared commitment to manage the burden of integrating a functional high-containment laboratory mechanism, clarifying empirical and ethical implications, and keeping transparency in policy deliberation process to conduct international BSL-4 R&D collaboration on the different contexts and locations in LMICs, in consideration of the current vulnerabilities of health infrastructure in LMICs.

## Author Contributions

YM conducted the literature search to retrieve the reference source information for analytical review of the contents and formulate the manuscript. RS conducted both supplemental information collection and selective expert interviews to assist YM in the conceptualization and articulation of the points of argument and in refining the draft manuscript. Both authors contributed to addressing the key messages in accordance with the trends and status revealed by the literature review.

### Conflict of Interest Statement

YM had consultancy works under the contract with Toray Industries, Inc., Japan, and RS is employed by Index Microbiology Ltd., UK. All financial relationships for these authors are outside the submitted work, and could not be construed as a potential conflict of interest.
