# Supplementary material for: Harmonization of Biosafety and Biosecurity Standards for High-Containment Facilities in Low- and Middle-Income Countries: An Approach From the Perspective of Occupational Safety and Health
Source: Front Public Health. 2019 Sep 12;7:249. doi: 10.3389/fpubh.2019.00249 (PMC6751378; doi:10.3389/fpubh.2019.00249)
Supplement: Supplementary file 1 [file Table_1.PDF]

Table 1. OSH points in the international R&D context indicated in the key biosafety-biosecurity policy documents (2004-2018)

Item	Year	Target/aim(s) (related to laboratory capacity development)	Target agent/disease(s)	Population/audiences mainly covered	Conceptual integration of the international R&D collaboration context	OSH in the international R&D collaboration context
**WHO Laboratory Biosafety Manual - 3rd Edition**	2004	Manual to guide an institution-based operational biosafety procedure	All infectious agents dealt with in public health laboratories	Laboratory managers, workers, and maintenance staff (national and local)	?	Generally requiring training for promotion of IPC awareness, preventive measures, and operational procedures
**WHO Responsible Life Sciences Research for Global Health Security**	2010	A guidance document to strengthen the culture of scientific integrity and excellence characterized by openness, honesty, accountability, and responsibility.	Mainly for emerging infectious diseases in the scope of IHR2005 and genetically modified, synthetic agents	Health researchers, laboratory managers and research institutions, potentially covering policy-makers, relevant national regulatory authorities, laboratory scientists and managers, and other professional members of scientific communities.	In generalized ways to encourage transparency, accountability, and trust for biosafety coordination from the technical viewpoint of capacity development	Outlining the measures to protect staff who report unlawful or irregular conduct from occupational detriment, as the elements of laboratory biorisk management systems.
**WHO Laboratory Biorisk Management Strategic Framework for Action 2012-2016**	2012	Under the IHR (2005), as the key activities for Governance, Norms, and Standards, countries are expected to raise awareness on the need to identify appropriate and proportionate regulatory controls and mechanisms for biorisk management, and develop model frameworks and approaches for their implementation in line with the vision for safe and secure environments in and around every laboratory in the world	Every possible infectious agent managed in the laboratories of any country	WHO member states for individual adaptation, relevant groups within WHO, WHO collaborating centers, biosafety associations and other key organizations such as OIE and FAO for partnership development.	Mentioned potential partnerships & collaboration between "internal and external experts" for leadership and communication	?
**WHO Laboratory Capacity Requirements for International Health Regulations**<br>**And their implementation in the WHO African Region (AFRO/WHO)**	2013	To identify approaches for laboratory capacity development to implement IHR in the WHO African), addressing the below 3 key questions: (a) What are the laboratory capacity requirements for IHR?(b) How do we ensure that stakeholders at the national level have a common understanding of the laboratory capacity requirements for IHR?(c) What are the approaches for developing and maintaining IHR laboratory capacity?	All IHR-targeted agents for laboratory diagnostics	Laboratory managers and professionals dealing with and overseeing laboratory services, as well as national authorities and stakeholders responsible for implementing IHR in all Member States in AFRO	Mentioned on international cooperation and partnerships addressed as encouraged for laboratory testing, that should be set up in advance so as to address the issues of specimen sharing and intellectual property rights.	?
**WHO Ebola virus disease (EVD)-Occupational Safety and Health: Joint WHO/ILO Briefing Note for Workers and Employers**	2014	Guidance/recommendation to enhance collaboration for IPC between employers and workers, as a briefing note for workers and employers in relation to EVD containment	EVD	Health workers at all levels of the health system – hospitals, clinics, laboratories, health posts, laundries, transport. Business travelers as the services recipient.	?	As a precondition for deployment of the treatment centers staffed by doctors, nurses who need to be properly qualified and equipped for their purposes.
**WHO Assessment Tool for Key Process associated with the Design, Construction, Operation, Maintenance and Regulation of BSL-3 Facilities in the WHO African Region**	2016	To meet the needs of the WHO AFRO Emerging and Dangerous Pathogen Laboratory Network (EDPLN) and provide information on the design, construction, and commissioning of biocontainment laboratories for the diagnosis of a range of emerging viral pathogens, setting the objectives mainly for the design, construction, and commissioning of BSL-3 facilities; and to provide a laboratory assessment tool to assess Emerging and Dangerous Pathogen Laboratories.	Those being diagnosed at BSL-3 laboratories to cover Marburg, Ebola, Lassa fever, Rift Valley fever, Lujo, Crimean-Congo hemorrhagic fever and Dengue viruses	Laboratory authorities & experts in AFRO	Outlining probable involvement of funding bodies and other institutions.	?

Item	Year	Target/aim(s) (related to laboratory capacity development)	Target agent/disease(s)	Population/audiences mainly covered	Conceptual integration of the international R&D collaboration context	OSH in the international R&D collaboration context
**Guidance for Managing Ethical Issues in Infectious Diseases Outbreaks**	2016	To outline the ethical principles that WHO believes should guide communication planning and implementation in the context of infectious disease outbreaks at every level – from frontline workers to policymakers. It makes the case for embedding ethics within the integrated global alert and response system for epidemics and other public health emergencies.	EVD and other epidemic-prone diseases	Government authorities, international community, community health workers, frontline response workers, foreign humanitarian aid workers	Described: obligations of governments and the international community; research during infectious diseases outbreak; frontline response workers' rights and obligations; and ethical issues in deploying foreign humanitarian aid workers	Equity and transparency – entities should ensure that risks are distributed among individuals and occupational categories in an equitable manner, and that the process of assigning workers is as transparent as possible
**GHSA Action Package**<br>**Prevent-3:** Biosafety and Biosecurity Assessment<br>An analysis of biosafety and biosecurity scores for WHO-JEE	2017	To provide tools and aid the development, implementation, and maintenance of national frameworks and systems for biosafety and biosecurity, country-specific legislation, laboratory licensing, and ensure pathogen control measures are in place as appropriate.	Emerging/re-emerging infectious diseases in the scope of IHR (2005)	Partners/countries	**?**	Only as components of biosecurity management plans for physical and personal security
**WHO Joint External Evaluation (JEE) Tool - 2nd Edition**<br>**Prevent:** Biosafety and Biosecurity<br>Detect: National Laboratory System/Workforce Development<br>Respond: Medical Countermeasures and Personnel Deployment	2018	The IHR (2005) is a binding international legal instrument in 194 countries, and the WHO-JEE is a voluntary, collaborative process to establish a baseline measurement of national capacity and capabilities.<br>▪To assess country capacity to prevent, detect, and rapidly respond to public health threats independently of whether they are naturally occurring, deliberate, or accidental, through the voluntary review process to identify the most urgent needs within their health security system<br>▪To measure country specific status and progress in achieving the targets	Emerging/re-emerging infectious diseases in the scope of IHR (2005)	Member states including external donor/partner countries	Medical countermeasures and personal deployment cover regional (international) collaboration to assist countries in overcoming the legal, logistical, and regulatory challenges to the deployment of public health and medical personnel from one country to another to achieve sustainable capacity for countries participating in a regional/international partnership or having a formal agreement with another country or international organization. (In the Respond Section)	Referred to only in the Glossary in line with the WHO Global Plan of Action (GPA) on Workers' Health (2008–17) and the ILO Promotional Framework for OSH Convention, 2006 (No. 187)
**WHO Occupational Safety and Health in Public Health Emergencies**	2018	A manual to provide technical guidance on good practices and procedures in establishing systems that can reduce occupational exposure, injury, illness, and death; decrease stress and reduce fears; and promote the health and well-being of healthcare and other response workers, particularly focused on needs in low-resource settings.	Infectious diseases (focus on EVD and cholera), natural disasters, radiological and chemical incidents, humanitarian conflicts	Health emergency response workers	**?**	Only covers incident-specific situations (Ebola, cholera)
